# Impact of the Cooking Process on Metabolite Profiling of *Acanthocereus tetragonus,* a Plant Traditionally Consumed in Mexico

**DOI:** 10.3390/molecules27123707

**Published:** 2022-06-09

**Authors:** Jaqueline Cornejo-Campos, Yenny Adriana Gómez-Aguirre, José Rodolfo Velázquez-Martínez, Oscar Javier Ramos-Herrera, Carolina Estefanía Chávez-Murillo, Francisco Cruz-Sosa, Carlos Areche, Emmanuel Cabañas-García

**Affiliations:** 1 Departamento de Química, Centro de Ciencias Básicas, Universidad Autónoma de Aguascalientes, Av. Universidad 940, Ciudad Universitaria, Aguascalientes 20100, Mexico; jaqui.1999.jcc@gmail.com; 2 CONACyT Research Fellow-Departamento de Química, Centro de Ciencias Básicas, Universidad Autónoma de Aguascalientes, Av. Universidad 940, Ciudad Universitaria, Aguascalientes 20100, Mexico; 3 División Académica de Ciencias Agropecuarias, Universidad Juárez Autónoma de Tabasco, Carretera Villahermosa-Teapa, km 25, Villahermosa 86280, Mexico; jrodolfovelazquez@gmail.com; 4 Unidad Profesional lnterdisciplinaria de Ingeniería, Campus Zacatecas, lnstituto Politécnico Nacional (UPllZ-lPN), Calle Circuito del Gato No. 202, Col. Ciudad Administrativa, Zacatecas 98160, Mexico; oramos@ipn.mx (O.J.R.-H.); cchavezm@ipn.mx (C.E.C.-M.); 5 Departamento de Biotecnología, Universidad Autónoma Metropolitana-Campus Iztapalapa, San Rafael Atlixco 186, Vicentina, Ciudad de México 09340, Mexico; cuhp@xanum.uam.mx; 6 Departamento de Química, Facultad de Ciencias, Universidad de Chile, Casilla 653, Santiago 7800024, Chile; areche@uchile.cl; 7 Centro de Estudios Científicos y Tecnológicos No. 18, Instituto Politécnico Nacional, Calle Circuito del Gato No. 202, Col. Ciudad Administrativa, Zacatecas 98160, Mexico

**Keywords:** marginalized communities, secondary metabolites, cacti, edible cactus, chromatography, alternative food

## Abstract

*Acanthocereus tetragonus* (L.) Hummelinck is used as an alternative food source in some Mexican communities. It has been shown that the young stems of *A. tetragonus* provide crude protein, fiber, and essential minerals for humans. In this work, we analyzed the phytochemical profile, the total phenolic content (TPC), and the antioxidant activity of cooked and crude samples of *A. tetragonus* to assess its functional metabolite contribution to humans. The phytochemical profile was analyzed using Ultra-High-Performance Liquid Chromatography coupled to High-Resolution Mass Spectrometry (UHPLC-PDA-HESI-Orbitrap-MS/MS). Under the proposed conditions, 35 metabolites were separated and tentatively identified. Of the separated metabolites, 16 occurred exclusively in cooked samples, 6 in crude samples, and 9 in both crude and cooked samples. Among the detected compounds, carboxylic acids, such as threonic, citric, and malic acids, phenolic acids, and glycosylated flavonoids (luteolin-O-rutinoside) were detected. The TPC and antioxidant activity were analyzed using the Folin–Ciocalteu method and the 2,2-diphenyl-1-picrylhydrazyl (DPPH) free radical inhibition method, respectively. The TPC and antioxidant activity were significantly reduced in the cooked samples. We found that some metabolites remained intact after the cooking process, suggesting that *A. tetragonus* represents a source of functional metabolites for people who consume this plant species.

## 1. Introduction

The arid and semi-arid zones of Mexico possess ecosystems with a high diversity of plants with potential applications; one of the main representative species from the arid Mexican environments are cacti species [1], which are traditionally used for food, traditional medicine, and construction [2]. It has been proposed that the Cactaceae family is native to the American continent, grouping around 2000 species, being Mexico the country with the highest diversity [3]. Cacti species are succulent plants that have suffered anatomical and physiological changes to adapt to the harsh environment [4] and whose morphology varies according to each genus and species. The Cactaceae Family includes four subfamilies, i.e., Opuntioideae, Pereskioideae, Cactoideae, and Maihuenioideae, and 63 genera [4]. It has been proposed that about 78% of cacti species are endemic; nevertheless, around 57% of the cactofloristic diversity is used by man for horticulture, food, medicine, forage, and handicrafts production [5]. Within the cacti family, the *Opuntia* genus is one of the most studied due to its nutritional interest.

Cacti species are characterized by the production of mucilage, which, in conjunction with different tissues, represents a source of functional metabolites for humans. Within the functional metabolites, phenolics have gained strong attention since they impact the quality of both fresh and processed plant-based foodstuffs, and their contribution to the maintenance of human health has been described [6]. Thus, analytical techniques such as liquid chromatography coupled with high-resolution mass spectrometry represent a tool for analyzing and discovering compounds with functional properties [7,8].

**Figure 1:**
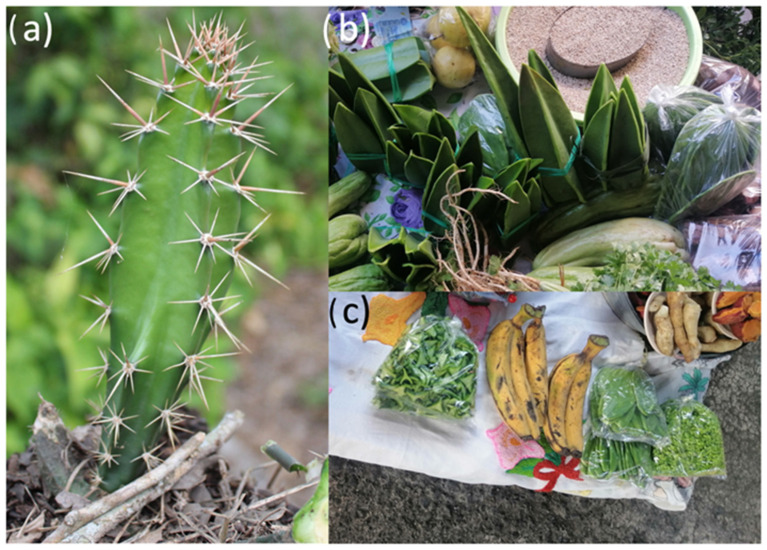
(**a**): Young stems of *Acanthocereus tetragonus* growing in the wild. (**b**,**c**): *A. tetragonus* and other vegetables produced by familiar farms and sold in local markets in northern Veracruz, México.

In Mexican gastronomy, the young stems of *Acanthocereus tetragonus*, traditionally known as cruzeta or jacube, are also used as a food source [9]. *A. tetragonus* is a columnar cactus distributed in coastal areas of the American continent in warm, sub-humid, and semi-arid regions [10,11]. *A. tetragonus* is traditionally collected from wild populations, and the young stems are sold at prices of around USD 1 per 150 g of fresh chopped young stems in local markets and urban venues (see Figure 1a–c), especially in the state of Veracruz, Mexico [12], where they represent a source of alternative food in some isolated regions. Regarding their chemical composition, it has been demonstrated that they are rich in proteins, fiber, and minerals such as phosphorus, potassium, magnesium, sodium, and copper, in addition to containing a high level of calcium [13]; nevertheless, as far as we know, no information exists regarding the phytochemical profile of *A. tetragonus*. In this study, we analyzed, by means of Ultra-High-Performance Liquid Chromatography coupled to High-Resolution Mass Spectrometry (UHPLC-PDA-HESI-Orbitrap-MS/MS), the phytochemical profile of cooked and crude samples of *A. tetragonus* to assess its functional metabolite contribution to the human diet.

## 2. Results and Discussion

### 2.1. Total Phenolic Content and Antioxidant Activity of Crude and Cooked Samples of A. tetragonus

Table 1 shows the results obtained for the total phenolic content and antioxidant activity of cooked and crude samples of *A. tetragonus.* The crude samples of *A. tetragonus* showed a higher phenolic content (40.79 ± 1.00 µg GAE per mg of DW) when compared with the cooked samples (27.52 ± 1.36 µg GAE per mg of DW). Statistically (*p* ≤ 0.05), there were significant differences between crude and cooked samples. Regarding the antioxidant activity, the crude samples showed the highest value (86.44%). Our results agree with findings for other plant species, such as *Prunus mahaleb* (Rosaceae), where the TPC and antioxidant activity were significantly reduced after heating treatment [14]. In this regard, for *Bixa orellana* (*Bixaceae*), it has been proposed that the thermal degradation rate of phenolic compounds depends on the temperature applied to the samples, and the degradation curve shows a first-order kinetic behavior [15]. Our evidence suggests that *A. tetragonus* TPC and antioxidant activity are reduced by the traditional food preparation process. Nevertheless, further investigations are required to evaluate the degradation rate of *A. tetragonus* compounds and the limits of temperature and exposure time within which food materials should be processed to ensure a minor degradation of the functional metabolites and a balance between their functional and sensory characteristics.

### 2.2. Phytochemical Analysis of A. tetragonus Young Stems

The methanolic extracts of crude and cooked stems of *A. tetragonus* were analyzed by UHPLC-PDA-HESI-Orbitrap-MS/MS. Under the chromatographic conditions, 35 metabolites were separated and tentatively identified. Of the separated metabolites, 16 occurred in cooked samples, 6 in crude samples, and 9 in both crude and cooked samples. Among the detected compounds, carboxylic acids, such as threonic, citric, and malic acids, phenolic acids, and glycosylated flavonoids (luteolin-O-rutinoside) were tentatively identified. All metabolites showed a mass accuracy below 5 ppm, except for compound **14**.

It has been proposed that the cooking process may lead to two main phenomena: (1) the degradation of phytocompounds and (2) an increase in the extractability of compounds. Thus, the degradation or enhanced extractability may vary according to: (1) the food material, (2) the processing parameters, and (3) the chemical nature of the compounds and their thermal stability [16]. In our research, compounds such as simple organic acids (citric acid), phenolic acids (sinapic and eucomic acid), and one flavonoid (luteolin rutinoside) remained present after the thermal process.

Compounds **1** (rt: 2.63 min), 2 (rt: 2.74 min), and 3 (rt: 3.10 min) were only detected in crude samples of *A. tetragonus* and corresponded to simple organic acids. Compound **1** was identified as threonic acid since the pseudomolecular ion at *m/z* 135.0293 yielded fragments at *m*/*z* 119.0350 and *m*/*z* 103.0390, which were generated due to the loss of one and two molecules of water, respectively. This compound has been proposed to have potential as an androgen-driven balding prevention agent [17]. On the other hand, compounds **2** and **3** were identified as 2-hydroxy-succinic acid (malic acid), as proposed by Ledesma-Escobar, et al. [18]. In addition to citric acid, these carboxylic acids are responsible for some fruits’ sourness [19]. Although these compounds may be present in concentrations below the detection limits in cooked samples, our results suggest that the cooking process led to the breakdown of these organic acids. For other systems, it has been demonstrated that the cooking process strongly influences the modification of the pH, mainly in association with the heating rate [20].

Citric acid (compound **5**) and three of its derivatives (compounds **4**, **6**, and **8**) were detected in both crude and cooked samples. Citric acid was identified as reported previously [18,21], and its derivatives were identified since the characteristic fragment ions of citric acid were detected within the mass spectrum (see Table 2). Citric acid is a highly demanded organic acid by the food and beverages, pharmaceutical, and personal care industries. In addition, despite being one of the most widely employed and industrially demanded compound, this carboxylic acid still has potential growth in the global market; thus, new sources and alternatives for its production need to be investigated [22]. On the other hand, compound **7** (rt: 11.41 min) was only detected in crude samples and was identified as piscidic acid, since the pseudomolecular ion at *m/z* 255.0506 yielded fragments at *m/z* 193.0498 ([M-H-CHO_2_-OH]^−^), 165.0549 ([M-H-C_2_H_3_O_3_-OH]^−^), and 149.0600 ([M-H-2CHO_2_-OH]^−^). Piscidic acid has been previously detected in other cacti species such as *Coryphantha macromeris* [23] and prickly pear cactus (*Opuntia ficus* indica) [24], and its occurrence in green vegetables has been proposed [25]. This metabolite has also been detected in *Opuntia ficus* indica cladodes and proposed as one of the metabolites that exert a photoprotective effect against UVA-induced oxidative stress in human keratinocytes [26].

**Figure 2:**
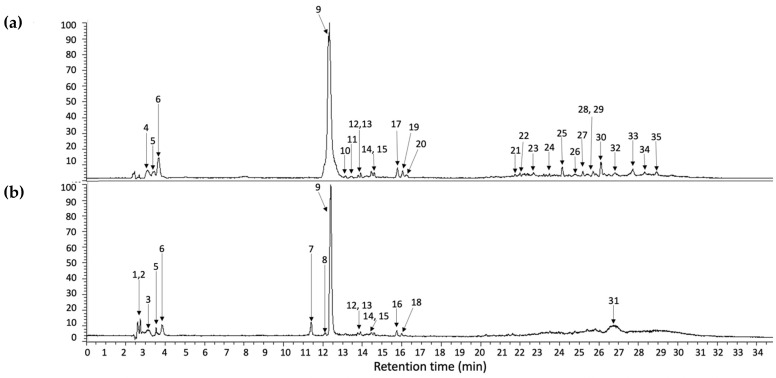
UHPLC chromatogram integrated at 254, 280, 320, and 440 nm of cooked (**a**) and crude (**b**) samples prepared from *A. tetragonus*. The peak numbers refer to those metabolites indicated in Table 1. The same numbers in “(**a**,**b**)” refer to metabolites found in both samples.

**Figure 3:**
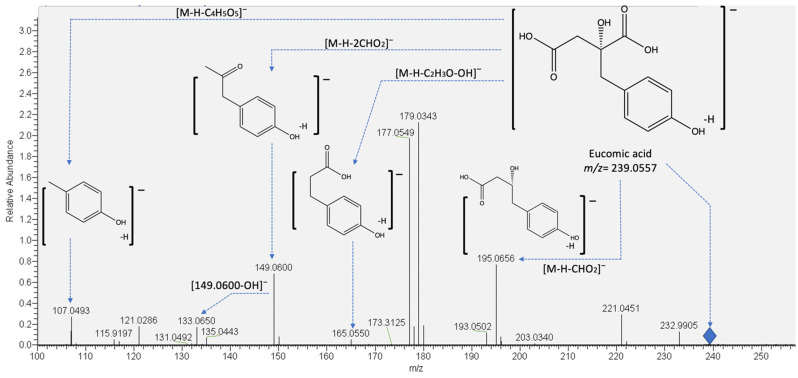
Proposed fragmentation pattern of eucomic acid (peaks 9 and 10).

On the other hand, compounds **9** and **10** were identified as eucomic acid isomers, as reported by Hernández, et al. [27]. This metabolite showed the highest relative abundance in the chromatogram (see Figure 2). For this compound, the pseudomolecular ion generated fragments at *m/z* 195.0656, 149.0600 133.0654, and 107.0502 (see Figure 3), and according to its relative abundance, it may correspond to one of the main metabolites present in crude and cooked samples of *A. tetragonus*, similar to that found for *Opuntia ficus* indica cladodes [28] and peel [29]. Eucomic acid has been previously reported in *Opuntia ficus* indica cladodes [30] and fruits [27] and as a key compound in leaf-opening processes in other plant species such as *Lotus japonicus* (Fabaceae) [31].

Compound **11** was identified as dihydroxybenzoic acid, as reported by Taamalli, et al. [32]. In our research, two flavonoid isomers were also detected. Compounds **12** and **13** were identified as luteolin-O-rutinoside, as proposed by García-Salas, et al. and Taamalli et al. [32,33], being the cleavage of the glycosidic moiety one of the first fragmentation steps followed by the cleavage of the C-C bond in the C ring of the basic flavonoid structure. Our results suggest that the consumption of *A. tetragonus* young stems contributes to luteolin derivative intake, since these isomers remained present in the samples after the thermal process. Further studies are required to evaluate the concentration of selected compounds after the heating or digestion processes. Luteolin-O-rutinoside has also been reported in other plants such as *Setaria italica* (Poaceae) [34] and in species of the genus *Cynara* (Asteraceae) [35]. Compound **14** is also of phenolic nature, and it was identified as diphenylphenol, since the pseudomolecular ion at *m/z* 245.0928 lost one water molecule, generating one fragment at *m/z* 229.1076. For this metabolite, no information exists regarding cacti species.

The occurrence of phenolic compounds and flavonoids has been previously reported in other edible cacti species such as *Opuntia humifusa* fruits [36] and cladodes [37] *Hylocereus polyrhizus* and *H. undatus* seeds [38], and *Opuntia* spp. [39]. For this group of metabolites, the antioxidant properties have been proposed [40,41], which can be influenced by the employed solvents and extraction processes. It has been proposed that the most polar solvents have the best extractive capacity [42,43]. Yahia and Mondragon-Jacobo [44] evaluated the antioxidant capacity of 10 varieties of *Opuntia* spp., using polar and nonpolar extractions. The polar extracts showed the highest biological activity. It has been demonstrated that the functional properties may also vary depending on the analyzed species [45] and can be affected by maturation process [46] and storage conditions [47] or vary in different sections of the plant [36,37].

In nature, compounds are usually found forming conjugates with carbohydrates. In this regard, for closely related plant species to *A. tetragonus*, De Leo, et al. [48] analyzed the phytochemical profile and flavonoid content of *Opuntia ficus* indica flowers, finding that glycosylated flavonoids such as isorhamnetin 3-O-robinobioside were present in high concentrations (42.69 mg/g), followed by isorhamnetin 3-O-galactoside (9.79 mg/g) and quercetin 3-O-ruthinoside (7.09 mg/g). Similarly, for *Opuntia ficus* indica and *Opuntia joconostle* byproducts, Mata, et al. [49] and Morales, et al. [50], respectively, found the presence of glycosylated phenolics, being the glycosylated derivatives of isorhamnetin the major compounds in *O. joconostle*. Jiménez-Aspee, et al. [51] evaluated the presence of metabolites in *Eulychinia acida* fruits, finding six main compounds, with glycosylated isorhamnetin in the highest concentration. This metabolite has also been detected in *Opuntia monacantha* extracts, as well as in kaempferol [52]. In our investigation, only one metabolite was a glycosylated flavonoid. Its relative abundance in the chromatogram was similar for crude and cooked samples, suggesting that this compound remains intact after the heating process.

Among the detected compounds, cinnamic and benzoic acids were also identified. Compounds **15**, **17**, and **19** corresponded to cinnamic acids: compounds **17** and **19** occurred exclusively in cooked samples and were identified as 4-hydroxycinnamic acid isomers since the pseudomolecular ion at *m/z* 163.0395 yielded one characteristic fragment ion at 119.0495 generated by the loss of CO_2_. On the other hand, compound **15** occurred in both cooked and crude samples and was identified as sinapic acid. For this metabolite, the pseudomolecular ion at *m/z* 223.0607 yielded fragments at *m/z* 193.0501 ([M-H-CH_3_O]^−^) and 179.0343 ([M-H-CH_3_O-CH_3_]^−^). Regarding the occurrence of benzoic acids in the samples, compound 20 was identified as gallic acid, as proposed by Zhang, et al. [53]. This metabolite has been reported in commercial and wild *Opuntia* species [54], and its commercial and potential industrial applications [55], as well as its pharmacological ability to suppress inflammatory responses in induced gastrointestinal disorders in mice [56], have been proposed.

Compound **23** was identified as di-tert-butyl 4-amino-4-(3-(tert-butoxy)-3-oxopropyl) heptanedioate. For this metabolite, the pseudomolecular ion yielded fragments at *m/z* 342.2284 ([M-H-C_4_H_9_O]^−^) and 270.1710 ([M-H-2C_4_H_9_O]^−^). Compound **24** was identified as 1,4-benzenediol, 2,2’-(6-dodecyne-1,12-diyl) bis [3,6-dimethoxy]^−^ since the pseudomolecular ion at *m/z* 501.2493 generated fragments at *m/z* 193.0867 ([C_11_H_13_O_3_]^−^). Compound **30** was identified as a linolenic acid derivative (Dirhamnosyl linolenic acid), as proposed by Li, et al. [57], and compound **33** as N,N-dihexyl-4-hydroxy-3,5-dimethoxybenzamide, since the parental ion at *m/z* 364.2494 yielded fragments at *m/z* 334.2388 ([M-H-CH_3_O]^−^) and at *m/z* 288.0328 ([M-H-2CH_3_O-OH]^−^). Similarly, compound **34** was identified as dodecyl 4-O-acetyl-2-O-(4-O-acetyl-6-deoxy-α-L-mannopyranosyl)-6-deoxy-β-D-galactopyranoside. For this metabolite, the pseudomolecular ion at *m/z* 561.3280 yielded fragments at *m/z* 515.3221, generated by the loss of two water molecules and one methyl group. As far as we know, this is the first time that these metabolites are reported in *A. tetragonus* and cacti species.

Compounds **21**, **22**, **26**–**32**, and **35** were not identified, since the spectrometric evidence did not match the literature’s information. This is interesting, since these metabolites may correspond to new unreported compounds. Furthermore, according to the UV absorption pattern of these compounds (ca. 275 nm), they may contain a phenolic ring within their structure. Our results indicate, for the first time, the metabolite profile of *A. tetragonus*; further studies are required to assess each compound’s total content and elucidate the structure of unknown metabolites.

## 3. Materials and Methods

### 3.1. Sample Collection and Preparation for Phytochemical Analysis

The young stems of *Acanthocereus tetragonus* were collected near the “Cerro de Horcones” community from the municipality of Alamo-Temapache, Veracruz, Mexico (21.073783, −97.782116) in November 2020. The collected samples were dethorned, and then the cuticle was removed manually. For the cooking process, each sample (2 kg) was sliced and boiled in water (1:3 p/v) at 90 °C for 15 min. Once the cooking process was achieved, the crude and cooked samples were dried in an oven at 40 °C (Gallenkamp, London, UK) for one week in dark conditions and then pulverized in a mortar. The resultant powder was subjected to extraction with methanol (three times, 1:3 p/v) in an ultrasonic bath (30 min each time). The resulting extract was filtered and concentrated by rotary evaporation (Heidolph Instruments, Schwabach, Germany) under reduced pressure at 40 °C. The samples were freeze-dried (FreeZone 4.5; Labconco Corporation, Kansas City, MO, USA), and each freeze-dried sample was resuspended (2.5 mg mL^−1^) in HPLC-Mass Spectrometry-grade methanol, sonicated for 10 min, filtered, and then used for the phytochemical analysis.

### 3.2. Phytochemical Analysis Using UHPLC-PDA-HESI-Orbitrap-MS/MS

Metabolite profiling was performed as reported previously [58,59,60], using a UHPLC system (Dionex™ UltiMate™ 3000; Thermo Fisher Scientific^®^, Waltham, MA, USA, hyphenated with a Thermo Scientific™ Q Exactive™ Focus Hybrid Quadrupole-Orbitrap™ mass spectrometer (Thermo Fisher Scientific^®^). The chromatographic system was equipped with a C18 column (ID: 150 × 4.6 mm, 5 µm; Restek Corporation, Bellefonte, PA, USA), a quaternary Series RS pump, and a Dionex™ UltiMate™ 3000 Series TCC-3000RS column compartments with an UltiMate™ 3000 Series WPS-3000RS autosampler (Thermo Fisher Scientific^®^). A Photodiode Array Detector (PDA) recording from 200 to 800 nm was also employed for peak characterization. The detection wavelengths were 254, 280, 320, and 440 nm for peak construction. The mobile phases consisted of a 1% formic aqueous solution (A) and acetonitrile (B). The elution program [time (min), %B] consisted of 5%B at time zero (0.00, 5), maintained for 5 min (5.00, 5), then %B was increased to 30% (10.00, 30) and maintained for 5 min (15.00, 30), further increased to 70% at min 20 (20.00, 70) and maintained for 5 min at the same proportion (25.00, 70); finally, the elution system returned to the initial conditions at min 35 (35.00, 5) and then was maintained for 12 min for column equilibration prior to each injection. The flow rate was set to 1.0 mL min^−1^, and the injection volume was 10 μL. The system was controlled by Thermo Scientific™ Chromeleon™ 7.2 Chromatography Data System (CDS) Software (Thermo Fisher Scientific^®^ and Dionex Softron GmbH division of Thermo Fisher Scientific^®^, Olching-Geiselbullach, Germany). The UHPLC was coupled to the mass spectrometer with a Heated Electrospray Ionization Source II (HESI II) (Thermo Fisher Scientific^®^). Nitrogen (purity > 99.999%; obtained from a Genius NM32LA nitrogen generator, Peak Scientific^®^) was employed as a collision and damping gas. As reported previously [58], the mass calibration for Orbitrap was performed weekly, in both negative and positive modes using caffeine and *N*-butylamine (Sigma-Aldrich^®^) as positive ions, and buspirone hydrochloride, sodium dodecyl sulfate, and taurocholic acid sodium salt, as negative ions. These compounds were dissolved in a mixture of acetic acid, acetonitrile, water, and methanol (Merck Darmstadt, Hesse, Germany) and infused using a Chemyx Fusion 100 syringe pump (Chemyx Inc., Stafford, TX, USA). Xcalibur™ 2.3 and TraceFinder™ 3.2 software (Thermo Fisher Scientific^®^) were used for UHPLC control and data processing. Q Exactive™ 2.0 SP2 (Thermo Fisher Scientific^®^) was used to control the mass spectrometer.

#### MS Parameters

HESI parameters were chosen as reported previously [58,59,60], using a sheath gas flow rate 75 units; an auxiliary gas unit, flow rate 20; capillary temperature 400 °C; auxiliary gas heater temperature 500 °C; spray voltage 2500 V (for ESI-); and S-Lens RF level 30. Full scan data in negative mode were acquired at a resolving power of 70,000 full width half maximum (FWHM) at *m*/*z* of 200. A scan range of *m*/*z* 100–1000 was chosen for the compounds of interest. The automatic gain control (AGC) was set at 3 × 10^6^, and the injection time was set to 200 milliseconds (ms). The scan rate was set at 2 scans s^−1^. The AGC target was set to 2 × 10^5^, with the maximum injection time of 20 ms. The precursor ions were filtered by the quadrupole operating at an isolation window of *m*/*z* of 2. The fore vacuum, high vacuum, and ultrahigh vacuum were maintained at approximately 2 mbar, from 105 to below 1010 mbar. Collision energy (HCD cell) was operated at 30 eV. Detection was based on the calculated exact mass and retention time of the target compounds. The mass tolerance window was set to 5 ppm.

### 3.3. Total Phenolic Compound Determination

Total phenolic compound determination was carried out by the Folin–Ciocalteu method described by Agbor, et al. [61]. A standard curve (*n* = 3) with gallic acid was prepared at different concentrations (0, 2, 4, 6, 8, 10, 12, and 14 µg/mL). Once the solution was prepared, 2.5 mL of deionized water and 0.1 mL of 1N Folin-Ciocalteu reagent (Sigma-Aldrich) were added to each tube. After 6 min, 0.25 mL of 10% aqueous sodium carbonate (Na_2_CO_3_) was added. After 30 min of reaction in dark conditions, the absorbance of the samples was monitored at 760 nm with a spectrophotometer (Jenway, Genova, Switzerland). The straight-line equation (y = 0.0159x − 0.0017) and the correlation coefficient (r^2^ = 0.9932) were obtained in Microsoft Excel version 16.60.

### 3.4. Antioxidant Activity

The antioxidant activity was determined using the 2,2-diphenyl-1-picrylhydrazyl (DPPH) free radical method proposed by Brand-Williams, et al. [62]. To 0.1 mL of the extract (each extract at a concentration of 10 mg mL^−1^), 3.9 mL of DPPH^+^ methanolic solution (60 µM) was added. After 30 min of reaction, the absorbance of the samples was measured at 510 nm. The results were expressed as percentage of inhibition of the DPPH^+^ radical, with the formula: % inhibition = (Ai − Af)/Ai × 100, where Ai is the absorbance of the blank, and Af is the absorbance of the sample.

### 3.5. Statistical Analysis

Statistical analysis was performed using SigmaPlot software (Systat software, version 11.0). Sample differences were determined using one-way ANOVA. Statistical significance of the means was considered at *p* ≤ 5%.

## 4. Conclusions

Under the proposed conditions, 35 metabolites were separated and tentatively identified. The cooking process affected the antioxidant activity as well as the phytochemical profile, leading to the formation of new compounds and the degradation of organic acids. Our results indicate that some compounds such as citric acid, eucomic acid, luteolin rutinoside, and sinapic acid remained present after the cooking process. Among the detected compounds, eucomic acid showed the highest relative abundance in both crude and cooked samples. To the best of our knowledge, the secondary metabolite profiling of cooked and crude samples of *A. tetragonus* is reported here for the first time, as well as their fragmentation pattern. Our results show evidence of the kind of metabolites that are consumed when young stems of *A. tetragonus* are collected from wild populations, cooked, and used as alternative food by humans. Additionally, our findings offer the basis for future investigations focused on the isolation and quantification of target compounds as well as on the evaluation of their biological activities.

## Figures and Tables

**Table 1:** Total phenolic content and antioxidant activity of cooked and crude samples of *A. tetragonus.* The values represent the mean (*n* = 3) ± standard deviation. Equal letters indicate no statistically significant differences (*p* ≤ 0.05).

*A. tetragonus* Sample	Total Phenolic Content Gallic Acid Equivalents (µg mg^−1^ DW)	DPPH Inhibition (%)
Crude	40.79 ± 1.00 ^a^	86.44
Cooked	27.52 ± 1.36 ^b^	5.96

**Table 2:** Metabolites identified in cooked and crude young stems of *A. tetragonus* by UHPLC-PDA-HESI-Orbitrap-MS/MS.

Peak	Retention Time (min.)	Uv Max	Tentative Identification	Elemental Composition [M-H]^−^	Theoretical Mass (*m/z*)	Measured Mass (*m/z*)	Accuracy (ppm)	MSn Ions	Sample
**1**	2.63	237	Threonic acid	C_4_H_7_O_5_^−^	135.0299	135.0294	3.70	119.0350, 103.0390	Crude
**2**	2.74	249	2-Hydroxy-Succinic Acid (malic acid)	C_4_H_5_O_5_^−^	133.0139	133.0134	3.76	115.0028	Crude
**3**	3.10	260	2-Hydroxy-Succinic Acid (malic acid) isomer	C_4_H_5_O_5_^−^	133.0139	133.0135	3.01	115.0028	Crude
**4**	3.12	262	Citric acid derivative	C_5_H_7_O_5_^−^	-	147.0293	-	111.0079	Cooked
**5**	3.51	265	Citric acid	C_6_H_7_O_7_^−^	191.01973	191.0193	2.25	111.0079	Crude, cooked
**6**	3.85	262	Citric acid derivative isomer	C_5_H_7_O_5_^−^	-	147.02916	-	111.0080	Crude, cooked
**7**	11.41	281	Piscidic acid	C_11_H_11_O_7_^−^	255.051	255.0506	1.57	193.0498, 165.0549, 149.0600	Crude
**8**	12.14	250	Citric acid derivative	C_7_H_11_O_5_^−^	175.0612	175.0606	3.43	111.0077	Crude
**9**	12.39	240, 280	Eucomic acid	C_11_H_11_O_6_^−^	239.0561	239.0557	1.67	195.0656, 165.0550, 149.0600, 133.0650, 107.0493	Crude, cooked
**10**	13.17	248, 285	Eucomic acid isomer	C_11_H_11_O_6_^−^	239.0561	239.0560	0.42	195.0656, 165.0550, 149.0600, 133.0650, 107.0493	Cooked
**11**	13.49	250	Dihydroxybenzoic acid	C_7_H_5_O_4_^−^	153.0188	153.0188	0.00	109.0287	Cooked
**12**	13.80	275, 360	Luteolin rutinoside	C_27_H_29_O_15_^−^	593.1518	593.1507	1.85	285.0399	Crude, cooked
**13**	13.91	275, 360	Luteolin rutinoside isomer	C_27_H_29_O_15_^−^	593.1518	593.1507	1.85	285.0399	Crude, cooked
**14**	14.44	280, 335	Diphenylphenol	C_18_H_13_O^−^	245.0972	245.0928	17.95	229.1076	Crude, cooked
**15**	14.58	255, 340	Sinapic acid	C_11_H_11_O_5_^−^	223.0607	223.0607	0.00	179.0708 193.0501	Crude, cooked
**16**	15.75	315	4-Hydroxycinnamic Acid isomer I	C_9_H_7_O_3_^−^	163.0396	163.0391	3.07	119.0493	Crude
**17**	15.82	315	4-Hydroxycinnamic Acid isomer II	C_9_H_7_O_3_^−^	163.0396	163.0400	2.45	119.0495	Cooked
**18**	15.99	315	4-Hydroxycinnamic Acid isomer III	C_9_H_7_O_3_^−^	163.0396	163.0400	2.45	119.0492	Cooked
**19**	16.05	325	4-Hydroxycinnamic Acid isomer IV	C_9_H_7_O_3_^−^	163.03958	163.03958	0.00	119.0495	Cooked
**20**	16.28	250	Gallic acid	C_7_H_5_O_5_^−^	169.0144	169.01369	4.20	125.0235	Cooked
**21**	21.8	278	Unknown	C_21_H_36_NO_6_^−^	-	398.2549	-	353.9409, 340.0991	Cooked
**22**	22.03	279	Unknown	C_22_H_38_NO_6_^−^	-	412.2705	-	291.0549, 252.1563	Cooked
**23**	22.72	260, 320	Di-tert-butyl 4-amino-4-(3-(tert-butoxy)-3-oxopropyl)heptanedioate	C_22_H_40_NO_6_^−^	414.2861	414.2861	0.00	344.2484, 270.1710	Cooked
**24**	23.52		1,4-Benzenediol, 2,2’-(6-dodecyne-1,12-diyl) bis [3,6-dimethoxy^•^]	C_28_H_37_O_8_^−^	501.2493	501.2493	0.00	193.0867	Cooked
**25**	24.20	285	linolenic acid derivative	C_30_H_53_N_6_O_14_^−^	-	721.3663	-	277.2169	Cooked
**26**	24.84	290	Unknown	Not given	-	562.3153	-	341.0167, 268.9643	Cooked
**27**	25.25	275	Unknown	C_30_H_55_N_6_O_14_^−^	-	723.3820	-	500.3238 302.3297 115.1763	Cooked
**28**	25.46	278	Unknown	C_29_H_41_N_2_O_11_^−^	-	593.2735	-	468.8837, 321.3711, 2423554	Cooked
**29**	25.74	270	Unknown	C_27_H_40_O_7_^−^	-	476.2781	-	226.2443	Cooked
**30**	26.15	260	Dirhamnosyl linolenic acid	C_28_H_47_O_11_^−^	559.3124	559.3124	0.00	277.2171	Cooked
**31**	26.81	275	Unknown	Not given	-	197.8075	-	-	Crude
**32**	26.86	265	Unknown	C_21_H_43_N_3_O_15_^−^	-	577.2690	-	499.1897, 434.2263, 283.1618	Cooked
**33**	27.78	254, 286	N,N-dihexyl-4-hydroxy-3,5-dimethoxybenzamide	C_21_H_34_NO_4_^−^	364.2493	364.2494	0.27	334.2388, 288.2328	Cooked
**34**	28.39	270	Dodecyl 4-O-acetyl-2-O-(4-O-acetyl-6-deoxy-α-L-mannopyranosyl)-6-deoxy-β-D-galactopyranoside	C_28_H_49_O_11_^−^	561.3280	561.3280	0.00	515.3221	Cooked
**35**	28.98	275	Unknown	C_19_H_38_O_7_^−^	-	378.2650	-	272.2274, 199.1150, 175.0970, 151.2393	Cooked

## Data Availability

Data is contained within the article.

## Sample Availability

Plant extracts are available from the authors.
